# Announcement

**Published:** 2015-02-27

## World Birth Defects Day — March 3, 2015

Every year an estimated 7.9 million infants (representing 6% of total births worldwide) are born with a serious birth defect (*1*). In many countries, birth defects are one of the leading causes of death in infants and young children (*2*). Infants who survive and live with these conditions are at an increased risk for long-term disabilities. For this event’s inaugural year, CDC is collaborating with 11 other organizations to implement World Birth Defects Day. The goals of this worldwide observance are to 1) increase global awareness about the occurrence of birth defects; 2) increase awareness of available treatment services; 3) expand referral and care services for all persons with birth defects; 4) increase implementation of primary prevention programs for birth defects; and 5) stimulate action among the public, members of governments, nongovernmental organizations, and health care providers to improve the care of affected children.

For World Birth Defects Day, CDC and its partners seek to build momentum for this initiative and together, work to increase birth defects surveillance capacity and expand prevention initiatives worldwide. CDC invites others to participate in World Birth Defects Day this year by sharing stories and information on social media using the hashtag #WorldBDDay. On March 3, all are encouraged to join the worldwide effort towards healthier women, healthier pregnancies, and healthier infants.
